# Association of Antibiotics Administration Timing With Mortality in Children With Sepsis in a Tertiary Care Hospital of a Developing Country

**DOI:** 10.3389/fped.2020.00566

**Published:** 2020-09-09

**Authors:** Alaa Alsadoon, Moudi Alhamwah, Bassam Alomar, Sara Alsubaiel, Adel F. Almutairi, Ramesh K. Vishwakarma, Nesrin Alharthy, Yasser M. Kazzaz

**Affiliations:** ^1^Department of Pediatrics, Ministry of National Guards—Health Affairs, Riyadh, Saudi Arabia; ^2^Pediatrics Emergency Department, Ministry of National Guards—Health Affairs, Riyadh, Saudi Arabia; ^3^King Saud bin Abdulaziz University for Health Sciences, Riyadh, Saudi Arabia; ^4^Science and Technology Unit, King Abdullah International Medical Research Center, Riyadh, Saudi Arabia; ^5^Department of Biostatistics and Bioinformatics, King Abdullah International Medical Research Center, Riyadh, Saudi Arabia; ^6^College of Applied Medical Sciences, King Saud bin Abdulaziz University for Health Sciences, Riyadh, Saudi Arabia; ^7^King Abdullah International Medical Research Center, Riyadh, Saudi Arabia; ^8^College of Medicine, King Saud bin Abdulaziz University for Health Sciences, Riyadh, Saudi Arabia

**Keywords:** Saudi Arabia, children, sepsis, septic shock, pediatric intensive care unit, sepsis resuscitation bundle

## Abstract

**Objective:** To investigate the association between antibiotics administration timing with morbidity and mortality in children with severe sepsis and septic shock, presenting to a tertiary care center in a developing country.

**Methods:** This is a retrospective study of children aged 14 years or younger diagnosed with severe sepsis or septic shock at a free-standing tertiary children's hospital in Saudi Arabia between April 2015 and February 2018. We investigated the association between antibiotic administration timing and pediatric intensive care unit (PICU) mortality, PICU length of stay (LOS), hospital LOS, and ventilation-free days after adjusting for confounders.

**Results:** Among the 189 admissions, 77 patients were admitted with septic shock and 112 with severe sepsis. Overall, the mortality rate was 16.9%. The overall median time from sepsis recognition to antibiotic administration was 105 min (IQR: 65–185.5 min); for septic shock patients, it was 85 min (IQR: 55–148 min), and for severe sepsis, 130 min (IQR: 75.5–199 min). Delayed antibiotic administration (> 3 h) was associated with 3.85 times higher PICU mortality (95% confidence intervals 1.032–14.374) in children with septic shock than in children who receive antibiotics within 3 h, after controlling for severity of illness, age, comorbidities, and volume resuscitation. However, delayed antibiotics administration was not significantly associated with higher PICU mortality in children diagnosed with severe sepsis.

**Conclusions:** Delayed antibiotics administration in children with septic shock admitted to a free-standing children's hospital in a developing country was associated with PICU mortality.

## Introduction

Sepsis is the most common cause of death in children (1). In developed countries, mortality rates because of severe sepsis and septic shock range from 10 to 50% (2–4). The prevalence of sepsis and septic shock continues to rise (5, 6). The increasing prevalence of sepsis, which is associated with poor outcomes, has led to the development of practice guidelines by several organizations. In 2002, the American College of Critical Care Medicine-Pediatric Advanced Life Support (ACCM-PALS) released the first clinical practice parameters for hemodynamic support of pediatric and neonatal shock. The clinical guidelines comprised rapid identification of sepsis, management with fluid resuscitation and antibiotic administration in the first hour, and intensive care hemodynamic support with source control (7). These guidelines were revised in 2007 and 2014 (8, 9). In addition, the Surviving Sepsis Campaign (SSC) published the first pediatric guidelines in 2020 (10).

The ACCM-PALS guidelines recommend antibiotic administration within 1 h of sepsis recognition (8). The 2020 SSC guidelines added a distinction between children with septic shock and severe sepsis, recommending antibiotic administration within 1 h in children with septic shock and within 3 h in children with severe sepsis, although with very low quality of evidence to support these recommendations (10). Unfortunately, the literature that examines the association between the timing of antibiotics administration and its outcomes in the pediatric population is scarce, with contradictory results (11–14). In addition, adult data cannot be extrapolated to children owing to several key developmental differences in the immune system response to infection and treatment (15, 16).

To the best of our knowledge, there are no epidemiological reports on sepsis in the pediatric population in Saudi Arabia. However, there are two reports addressing sepsis management. Hasan et al. (17) reported on a retrospective quality initiative implementing sepsis guidelines in a pediatric intensive care unit (PICU), which was evaluated over an 8-month period with 65 patients and a mortality rate of 26–47%. However, adherence rates for time-sensitive interventions and the relationship between adherence and outcomes were not reported (17). In a national survey conducted by Thabet et al. (18) a high level of adherence to the management guidelines among intensivists was found. Nonetheless, this study depicts the respondents' perceptions and not real-life practices.

The goal of the present study was to investigate the association between antibiotic administration timing with morbidity and mortality in children with severe sepsis and septic shock.

## Materials and Methods

### Patients

This retrospective cohort study was conducted at the King Abdullah Specialist Children's Hospital, a government tertiary and academic free-standing hospital in Riyadh, Saudi Arabia. The hospital's inpatient bed capacity is ~220, with 25 beds assigned for closed medical-surgical PICU. The unit's patient case-mix include general surgery, neurosurgery, solid organ transplant, bone marrow transplant, and trauma. The hospital was accredited by the Joint Commission International.

Patients aged 0–14 years admitted between April 2015 and January 2018 were included in the study if they met the criteria for severe sepsis or septic shock as defined by the International Pediatric Sepsis Consensus Conference (IPSCC) (19). Patients were identified using the PICU administrative database and medical records with International Classification of Diseases (ICD-10) sepsis-related codes (20).

**Figure 1:**
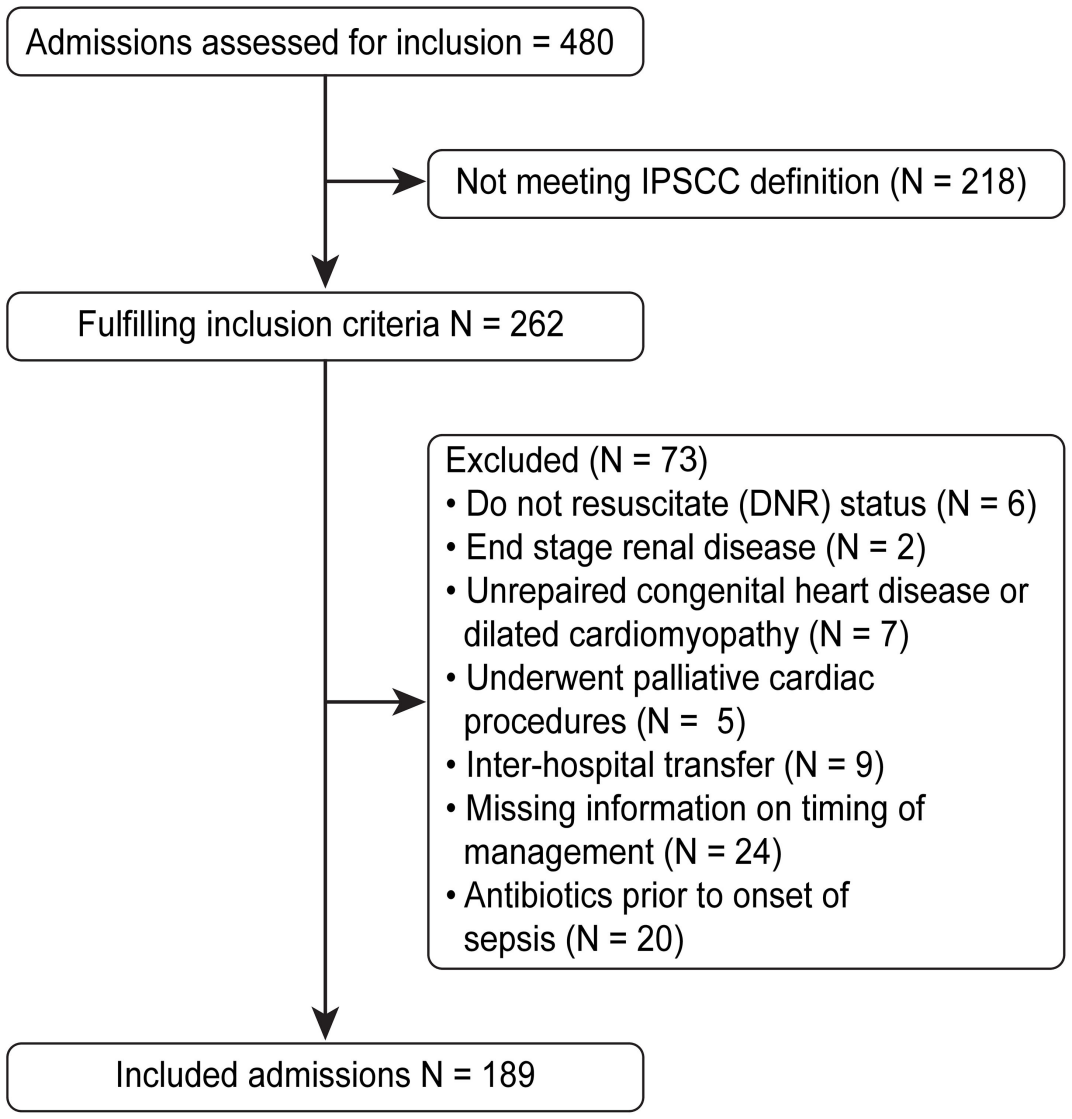
Recruitment flow chart.

Children were excluded if they were diagnosed with an illness that might compromise the “volume resuscitation” component of the guidelines, which includes end stage renal disease, unrepaired congenital heart disease or dilated cardiomyopathy, or palliative cardiac procedures. In addition, children with do-not-resuscitate status, inter-hospital transferees after identification of sepsis and initiation of management, and children who were on antibiotics prior to the onset of sepsis were excluded. A total of 480 admissions were reviewed, of which 189 were included in the analysis (Figure 1). The study was approved by the Institutional Review Board at the King Abdullah International Medical Research Center, and the requirement for informed consent was waived.

All the data were individually obtained from electronic medical records and were manually reviewed. Variables collected included demographic data, Pediatric Index of Mortality 3 (PIM 3) score (21), comorbidities, organ dysfunction on sepsis recognition, hospital length of stay (LOS), PICU LOS, management data (fluid resuscitation, antimicrobial therapy, and mechanical ventilation), source of infection, and culture results.

Sepsis recognition time varied depending on the area of presentation. For emergency room patients (ER), it was triage time (22); for inpatients, it was the time the patient was first recognized to have severe sepsis based on physicians' or nurses' documentation, or according to the IPSCC definitions for severe sepsis and septic shock—whichever was present or documented first (Supplementary Table 1) (19). The source of infection was determined based on the primary site of infection according to physicians' diagnosis based on positive cultures, positive polymerase chain reaction, or radiologic imaging findings. The definition of “organ dysfunction” was based on IPSCC criteria (19). Hospital acquired infection was defined as an infection developing in a patient hospitalized for 48 h or more before the onset of signs and symptoms consistent with the infection (23).

### Data Analysis and Management

IBM SPSS version 26 was used to analyze the data. The median and percentile (Q1–Q3) values were used to describe the quantitative variables of age, PIM 3 score, and organ dysfunction. Frequencies and percentages were used to describe the categorical variables, such as gender, comorbidities, source of infection, and antibiotics administration timing within 1, 2, and 3 h.

The primary analysis was the association between the timing of antibiotic administration with PICU mortality of children with septic shock and severe sepsis. Other outcomes included PICU LOS, hospital LOS, and ventilation-free days (VFD). VFD was defined as the number of days between successful extubation and day 28; thus, it is considered zero if the patient dies before 28 days. Based on previous findings, the predefined variables of interest were the time from meeting the sepsis criteria to the antibiotic administration at 1, 2, and 3 h and the volume of boluses in the first 2 h (11, 13, 14). Univariate comparisons using the Fisher's exact test and Mann-Whitney *U-*test for non-parametric data were used for categorical and continuous variables, respectively. The associations identified in the analyses were expressed as odds ratios (OR) and 95% confidence intervals (CI).

Multivariable logistic regression was used for assessing the timing of antibiotic administration with PICU mortality in the septic shock and severe sepsis subgroups. Finally, we built multivariate linear regression models for evaluating the impact of antibiotic administration within 1 h on continuous outcomes (PICU LOS, hospital LOS, and VFD). Since PICU LOS, hospital LOS, and VFD were not normally distributed, data were log-transformed prior to the statistical modeling. Age, comorbidities, severity of illness (PIM 3 score), and volume of boluses in the first 2 h were included in the regression analysis based on previous findings (12, 13). A *P* < 0.05 was considered statistically significant for all analyses.

## Results

During the study period, there were a total of 172 patients with 189 admissions meeting the inclusion and exclusion criteria. There was almost equal gender distribution, with 86 males (45.5%) and the median age was 19 months (IQR: 3–74.5 months). The median PICU LOS was 8 days (IQR: 2–23.5 days), and the median hospital LOS was 22 days (IQR: 9–30 days). Among the participants, 79.9% (*n* = 155) were known to have several comorbidities. The overall PICU mortality rate was 16.9% (*n* = 32).

Table 1 shows the characteristics of patients in the cohort. Two-fifths of the children (*n* = 77, 40.7%) had septic shock. The sources of infection were primarily respiratory (*n* = 107, 56.6%), followed by bloodstream (*n* = 20, 10.6%). Cultures were positive in 72 children. Almost half of the children (*n* = 108, 57.1%) had the onset of sepsis (time zero) in the ER, while the remainder (*n* = 81, 42.9%) developed sepsis while admitted in the hospital wards. Forty four children (23.3%) were diagnosed with hospital-acquired infections. Children with septic shock had more positive cultures, more multiorgan dysfunction syndrome on sepsis recognition, and lower VFD than children with severe sepsis (all *P* < 0.05)

**Table 1:** Characteristics of the study cohort.

		**All patients (*n =* 189)**		**Septic shock (*n =* 77)**		**Severe sepsis (*n =* 112)**		
	**Variable**	**N**	**%**	**N**	**%**	**N**	**%**	***p***
Gender	Male	86	45.5%	34	44.2%	52	46.4%	0.437
Source of infection	Respiratory	107	56.60%	32	41.60%	75	67.00%	0.004
	Blood stream	20	10.60%	14	18.20%	6	5.40%	
	Abdominal	17	9.00%	6	7.80%	11	9.80%	
	Genitourinary	10	5.30%	4	5.20%	6	5.40%	
	Skin/Soft tissue	6	3.20%	5	6.50%	1	0.90%	
	CNS	3	1.60%	2	2.60%	1	0.90%	
	Endocarditis	1	0.50%	1	1.30%	0	0.00%	
	Unknown	25	13.20%	13	16.90%	12	10.70%	
Positive culture	Yes	72	38.1%	36	46.8%	36	32.1%	0.030
Comorbidities	Yes	151	79.9%	64	83.1%	87	77.7%	0.233
Mechanical ventilation	Yes	106	56.1%	63	81.8%	43	38.4%	0.000
Mortality	Yes	32	16.9%	15	19.5%	17	15.2%	0.280
	Hospital-acquired infection	44	23.3%	16	20.8%	28	25%	0.310
		**Median**	**IQR**	**Median**	**IQR**	**Median**	**IQR**	
	MODS	3	2–4%	3	3–4	2	1–3%	0.000
	PIM 3	3.97%	1.46–3.99%	5.54%	1.46–15.3%	3.63%	1.45–10.07%	0.155
	Age (months)	19	3–74.5%	18	3–92	19.5	4.25–68.75%	0.744
	PICU LOS (days)	8	2–23.5%	10	4.5–28	6.5	2–17%	0.050
	Hospital LOS (days)	22	9–30%	30	10–30	19	9–30%	0.085
	VFD (days)	23	0–28%	0	0–23	28	17.5–28%	0.000

*CNS, central nervous system; MODS, multiorgan dysfunction syndrome; PIM, Pediatric Index of Mortality; LOS, length of stay*.

**Figure 2:**
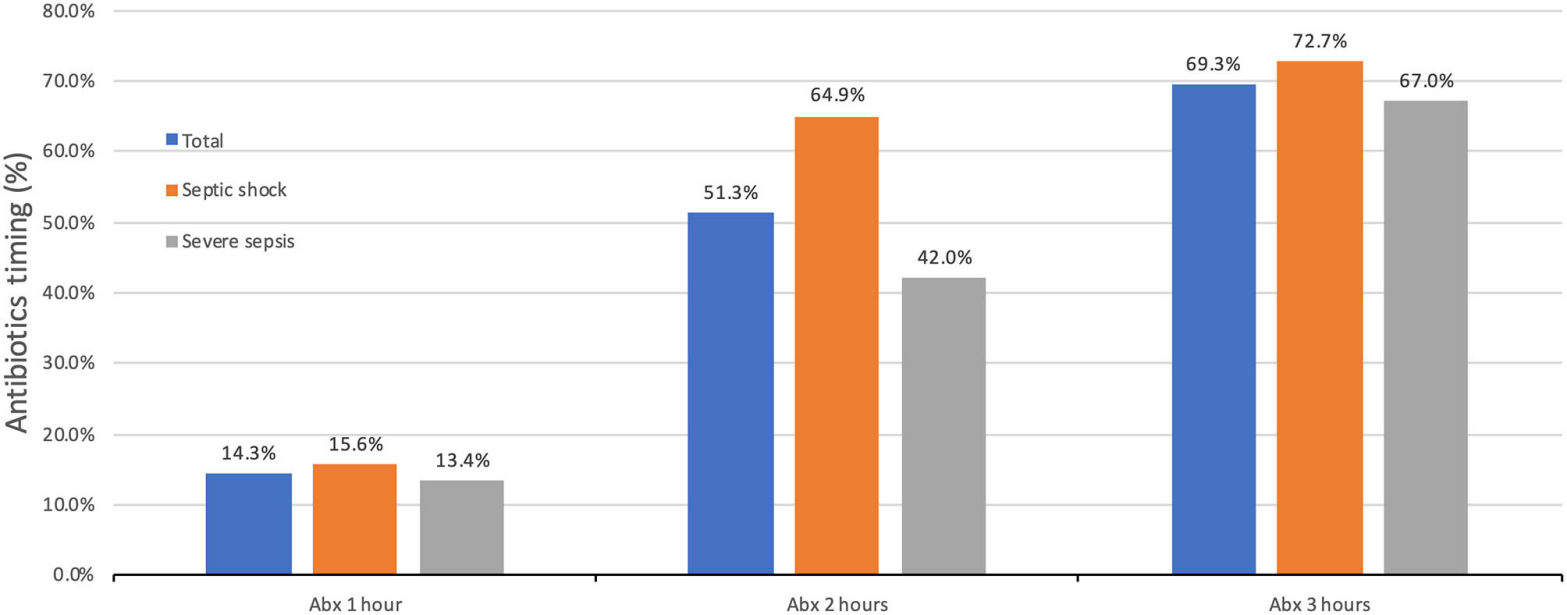
Percentage of children who received antibiotics within 1, 2, and 3 h.

The percentage of children who received antibiotics within 1 h of the onset of sepsis was 14.3% (*n* = 27 out of 189), while 51.3% (*n* = 97 out of 189) received antibiotics within 2 h (Figure 2). The overall median time from sepsis recognition to antibiotic administration was 105 min (IQR: 65–185.5 min); for septic shock patients, it was 85 min (IQR: 55–148 min), and 130 min (IQR: 75.5–199 min) for severe sepsis.

Tables 2, 3 summarize the OR and adjusted odds ratios (AOR) for PICU mortality associated with antibiotics administration timing beyond 1, 2, and 3 h for patients with septic shock and severe sepsis. In all patients, there was no difference in the mortality with antibiotics administration timing at 1, 2, or 3 h. At 3 h mark the mortality rate in children receiving antibiotics within 3 h and more than 3 h was 14.5 and 22.4%, respectively. Nonetheless, this was not statistically significant (*p* = 0.131). In patients with septic shock, there was an increased risk of mortality for each hour of delay, which became more predominant at the 3-h mark. There was a 14.3% mortality rate in children receiving antibiotics within 3 h and a 33.3% mortality rate in children receiving antibiotics after more than 3 h. However, this was not statistically significant (*p* = 0.063). In the severe sepsis cohort, there was no difference in the mortality with antibiotics administration timing at 1, 2, or 3 h. After adjusting for the age, comorbidities, severity of illness (PIM 3 score), and volume of boluses ≥ 20 mL/kg in the first 2 h from sepsis recognition (time zero), the AOR for PICU mortality was 3.85 times higher in children with septic shock and with antibiotics administration beyond 3 h in comparison to within 3 h (*p* = 0.045, 95% CI 1.032–14.374) (Table 3) (Supplementary Tables 2, 3 for AOR and effect sizes for all covariates in the models).

**Table 2:** PICU mortality by timing of antibiotics administration.

	**Administration time of initial antibiotics (h)**	**No. of patients**	**Mortality (n)**	**Mortality (%)**	**OR**	**95% CI**	***p***
All patients	≤ 1	27	4	14.8%	1.025	0.886–1.186	0.502
	>1	162	28	16.3%			
	≤ 2	97	16	16.5%	1.033	0.705–1.514	0.511
	>2	92	16	17.4%			
	≤ 3	131	19	14.5%	1.417	0.872–2.305	0.131
	>3	58	13	22.4%			
Septic shock	≤ 1	12	1	8.30%	1.135	0.95–1.356	0.266
	>1	65	14	21.5%			
	≤ 2	50	8	16%	1.447	0.755–2.772	0.225
	>2	27	7	25.9%			
	≤ 3	56	8	14.3%	2.067	1.015–4.207	0.063
	>3	21	7	33.3%			
Severe sepsis	≤ 1	15	3	20%	0.943	0.747–1.190	0.406
	>1	97	14	14.4%			
	≤ 2	47	8	17%	0.989	0.556–1.449	0.419
	>2	65	9	13.8%			
	≤ 3	75	11	14.7%	1.082	0.534–2.190	0.517
	>3	37	6	16.2%			

*OR, odds ratio; CI, confidence interval %; PICU, pediatric intensive care unit*.

**Table 3:** Multivariate analysis of the association of the timing of antibiotics administration with PICU mortality.

	**Variable**	**AOR**	**95% CI**	***p***
All patients	1-h delay	1.205	0.379–3.833	0.752
	2-h delay	1.208	0.550–2.651	0.638
	3-h delay	1.798	0.800–4.042	0.156
Septic shock	1-h delay	5.144	0.534–49.544	0.156
	2-h delay	2.181	0.632–7.532	0.217
	3-h delay	3.852	1.032–14.374	0.045
Severe sepsis	1-h delay	0.613	0.149–2.515	0.497
	2-h delay	0.973	0.320–2.961	0.962
	3-h delay	1.365	0.433–4.302	0.595

*Adjusted variables: PIM 3 score, age, comorbidities, and volume of resuscitation in the first 2 h. AOR, adjusted odds ratio; CI, confidence interval; PICU, pediatric intensive care unit*.

Our multivariate linear regression models (Table 4) demonstrated that administering antibiotics within 1 h was independently associated with shorter PICU LOS for patients with severe sepsis (0.32 days, 95% CI 0.607–0.049 days) in comparison to beyond 1 h. For septic shock cohort, we observed longer PICU LOS (0.31 days, 95% CI 0.020–0.603 days) in comparison to beyond 1 h.

**Table 4:** Multivariate linear regression for PICU LOS, hospital LOS, and VFD.

**Antibiotics within 1 h**	**Effect on increasing/decreasing duration**								
	**PICU LOS**			**Hospital LOS**			**VFD**		
	**Effect size**	**95% CI**	***P***	**Effect size**	**95% CI**	***p***	**Effect size**	**95% CI**	***p***
All patients	−0.055	−0.261 to 0.151	0.598	0.010	−0.139 to 0.160	0.892	0.021	−0.036 to 0.079	0.468
Septic shock	0.314	0.020 to 0.603	0.037	0.159	−0.099 to 0.417	0.223	0.026	−0.105 to 0.158	0.685
Severe sepsis	−0.328	−0.607 to −0.049	0.022	−0.074	−0.257 to 0.108	0.422	0.032	−0.016 to 0.080	0.190

*Adjusted variables: PIM 3 score, age, comorbidities, volume resuscitation in the first 2 h. VFD, ventilation-free days; LOS, length of stay; PICU, pediatric intensive care unit; CI, confidence interval*.

## Discussion

The main purpose of this study was investigating the association between the timing of antibiotics administration with PICU mortality, PICU LOS, hospital LOS, and VFD in the pediatric population admitted to a tertiary care center in Saudi Arabia. Our findings suggest the following: First, mere adherence to antibiotics administration within 1 h was insufficient. Second, in patients with septic shock, administration of antibiotics beyond 3 h from the recognition of sepsis was independently associated with a higher mortality rate. Third, the administration of antibiotics within 1 h in patients with septic shock was independently associated with longer PICU LOS; however, it was independently associated with shorter PICU LOS in children with severe sepsis.

In agreement with the global epidemiology of pediatric severe sepsis point prevalence study, our findings indicate that 79.9% of children with severe sepsis or septic shock had comorbidities, the most common source of infection was respiratory (24). The median age was 19 months, this age is lower than reported in the point prevalence study of 36 months of age. Nonetheless this was similar to the reported median age of 18 months in previous study reported from Saudi Arabia (17). This might be partially due to the limit of pediatric age group up to 14 years in Saudi Arabia.

Our study showed that in our hospital, adherence to antibiotic administration timing with sepsis practice guidelines was insufficient. Previous studies evaluating adherence to practice guidelines have shown similar data. Our median time to administer antibiotics was 105 min (IQR: 65–185.5 min), while the reported median times from Boston Children's Hospital, Stollery Children's Hospital, Children's Hospital of San Antonio, and Children's Hospital of Philadelphia were 96, 115, 135, and 140 min, respectively (11, 13, 14, 25).

Administering antibiotics beyond 3 h from sepsis recognition in children with septic shock was found to be an independent risk factor for mortality. The available literature addressing the association between rapid antibiotic administration and mortality is scarce. Our findings are similar to those reported by Weiss et al. (14) which involves a retrospective cohort of 130 patients, of which 27 had severe sepsis, and 103 had septic shock, with 3.92 OR for PICU mortality for children who received antibiotics beyond 3 h from sepsis recognition.

Although the 2020 SSC guidelines recommend starting antibiotics within 1 h from the recognition of septic shock and within 3 h of severe sepsis patients, we have only been able to show a statistically significant difference in mortality at 3 h from sepsis recognition (10). Several studies were not able to support the practice of antibiotic administration within 1 h as well. In a cohort of 1,179 patients with sepsis and septic shock who were treated according to the mandated sepsis protocol from 54 hospitals in New York State, Evans et al. (12) reported no association between initiating antibiotics administration within 1 h from sepsis recognition and mortality reduction. Nonetheless, the heterogeneity among the children with severe sepsis and septic shock might have underestimated the effect of early antibiotics (12). Creedon et al. (11) reported higher mortality in children who received antibiotics within 1 h from sepsis recognition in the ER (*p* = 0.009); however, the patients who received early antibiotics might have been sicker, and this might not have been captured by their risk adjustment tool. van Paridon et al. (13) found no association between early antibiotics administration and mortality for a cohort of 83 patients enrolled prospectively in Alberta Sepsis Network. Due to methodological variations, recommendations cannot simply be extrapolated based on the available studies.

Our study demonstrated statistically significant shorter PICU LOS in children with severe sepsis who received antibiotics within 1 h. Evans et al. (12) showed similar findings of shorter LOS among children with severe sepsis who adhered to sepsis practice guidelines. Paul et al. (26) reported that adherence to practice guidelines, including early antibiotic administration and fluid resuscitation, was associated with reduced PICU LOS in children with severe sepsis and septic shock.

This study has several limitations. First, this is an observational study, which makes it prone to bias and is not always able to establish causation. Second, this study is based on the data from a single center, which resulted in a relatively small sample size, and also, the difference in national health care systems, population demographics, resources, and staffing characteristics is a potential limitation to its generalizability. Lastly, we were unable to determine appropriate antibiotics because of the practical constraints and retrospective nature of this study. Nonetheless, our study strengthens the observation from the limited number of studies that delayed antibiotic administration is associated with higher mortality.

## Conclusions

In summary, our findings are in keeping with some available literature in pediatrics, which shows that adherence to antibiotics administration timing is low. In our hospital, a delay in administering antibiotics to children with septic shock is an independent risk factor for mortality and is associated with longer PICU LOS in children with severe sepsis. Further prospective research in the pediatric population with sepsis is essential to confirm the beneficial impact of antibiotic timing on patient outcomes.

## Data Availability Statement

The datasets used analyzed during the current study are available from the corresponding author upon reasonable request.

## Ethics Statement

The studies involving human participants were reviewed and approved by King Abdullah International Medical Research Center. Written informed consent from the participants' legal guardian/next of kin was not required to participate in this study in accordance with the national legislation and the institutional requirements.

## Author Contributions

YK: conceptualization, methodology, formal analysis, investigation, data curation, writing original draft, visualization, and supervision. AA: conceptualization, investigation, and writing original draft. NA: conceptualization, methodology, writing, review, and editing. MA and BA: investigation and writing original draft. SA: writing original draft. AFA and RV: formal analysis. All authors contributed to the article and approved the submitted version.

## Conflict of Interest

The authors declare that the research was conducted in the absence of any commercial or financial relationships that could be construed as a potential conflict of interest.
