# Supplementary material for: Association of Antibiotics Administration Timing With Mortality in Children With Sepsis in a Tertiary Care Hospital of a Developing Country
Source: Front Pediatr. 2020 Sep 9;8:566. doi: 10.3389/fped.2020.00566 (PMC7509148; doi:10.3389/fped.2020.00566)
Supplement: Supplementary file 1 [file Table_1.DOCX]

Table S1: Sepsis recognition time “Time Zero” definition

| Location of diagnosis | Definition |
| --- | --- |
| Emergency Room ER | Time of triage |
| Inpatient | Earliest sepsis related documentation or intervention including:   - Vital signs changed including 1st description of delayed perfusion (decreased peripheral pulses, mottled or cool extremities, capillary refill time (CRT) <1 or >3 sec, delayed or brisk), hypotension, altered mental status, oliguria <1ml/kg/hr - Physician’s documentation in patient’s chart. - Nurses documentation of discussion with physician for sepsis. - 1st orders for Sepsis (Antibiotics, cultures, fluid boluses, lactic acid). - Consultation to PICU for sepsis. - Abnormal lab results that meet severe sepsis criteria, if none of the previous were mentioned. |

Table S2: Multivariate analysis of the association of the timing of antibiotics administration with PICU mortality.

|  | Variable | 1-hour delay | | | 2-hour delay | | | 3-hour delay | | |
| --- | --- | --- | --- | --- | --- | --- | --- | --- | --- | --- |
|  |  | AOR | 95% CI | *p* | AOR | 95% CI | *p* | AOR | 95% CI | *p* |
| All cohort | Antibiotics delay | 0.83 | 0.261–2.639 | 0.752 | 0.828 | 0.377–1.818 | 0.638 | 1.798 | 0.8–4.042 | 0.156 |
|  | Age (months) | 0.995 | 0.987–1.003 | 0.232 | 0.995 | 0.987–1.003 | 0.243 | 0.996 | 0.988–1.004 | 0.305 |
|  | PIM 3 | 0.338 | 0.075–1.519 | 0.157 | 0.308 | 0.067–1.423 | 0.131 | 0.294 | 0.064–1.35 | 0.115 |
|  | Comorbidities | 1.15 | 0.411–3.22 | 0.79 | 1.147 | 0.41–3.208 | 0.793 | 1.16 | 0.413–3.256 | 0.778 |
|  | Volume in the first 2 hours | 0.942 | 0.418–2.123 | 0.885 | 0.953 | 0.422–2.153 | 0.908 | 0.999 | 0.438–2.274 | 0.997 |
| Septic shock | Antibiotics delay | 5.144 | 0.534–49.544 | 0.156 | 2.181 | 0.632–7.532 | 0.217 | 3.852 | 1.032–14.374 | 0.045 |
|  | Age (months) | 0.988 | 0.976–0.999 | 0.04 | 0.99 | 0.979–1.001 | 0.076 | 0.989 | 0.978–1.001 | 0.065 |
|  | PIM 3 | 0.585 | 0.047–7.244 | 0.676 | 0.494 | 0.04–6.133 | 0.583 | 0.587 | 0.042–8.142 | 0.692 |
|  | Comorbidities | 2.936 | 0.552–15.605 | 0.206 | 2.839 | 0.534–15.111 | 0.221 | 3.402 | 0.612–18.916 | 0.162 |
|  | Volume in the first 2 hours | 0.426 | 0.092–1.98 | 0.276 | 0.472 | 0.101–2.22 | 0.342 | 0.542 | 0.114–2.575 | 0.441 |
| Severe Sepsis | Antibiotics delay | 0.613 | 0.149–2.515 | 0.497 | 0.973 | 0.32–2.961 | 0.962 | 1.365 | 0.433–4.302 | 0.595 |
|  | Age (months) | 1.001 | 0.988–1.013 | 0.886 | 1.001 | 0.989–1.013 | 0.9 | 1.001 | 0.989–1.014 | 0.825 |
|  | PIM 3 | 0.297 | 0.039–2.259 | 0.241 | 0.332 | 0.041–2.713 | 0.304 | 0.289 | 0.037–2.244 | 0.235 |
|  | Comorbidities | 0.761 | 0.188–3.083 | 0.702 | 0.774 | 0.192–3.124 | 0.719 | 0.753 | 0.185–3.059 | 0.692 |
|  | Volume in the first 2 hours | 1.295 | 0.451–3.715 | 0.631 | 1.315 | 0.457–3.783 | 0.611 | 1.352 | 0.47–3.888 | 0.576 |

AOR: adjusted odds ratio, CI: confidence interval, PICU: pediatric intensive care unit, PIM 3: Pediatric Index of Mortality-3

**Table S3.** Multivariate linear regression for PICU LOS, hospital LOS, and VFD.

|  | Variable | Effect on increasing/decreasing duration | | | | | | | | |
| --- | --- | --- | --- | --- | --- | --- | --- | --- | --- | --- |
|  |  | PICU LOS | | | Hospital LOS | | | VFD | | |
|  |  | Effect size | 95% CI | *P* | Effect size | 95% CI | *p* | Effect size | 95% CI | *p* |
| All cohort | Abx within 1 hr | -0.055 | -0.261–0.151 | 0.598 | 0.010 | -0.139–0.16 | 0.892 | 0.021 | -0.036–0.079 | 0.468 |
|  | Age (months) | -0.001 | -0.002–0.001 | 0.472 | -0.001 | -0.002–0.000 | 0.184 | 0.001 | 0.000–0.001 | 0.004 |
|  | PIM 3 | 0.305 | -0.01–0.619 | 0.057 | 0.303 | 0.068–0.539 | 0.012 | -0.019 | -0.109–0.072 | 0.684 |
|  | Comorbidities | -0.103 | -0.289–0.082 | 0.273 | -0.152 | -0.287–-0.017 | 0.028 | 0.036 | -0.015–0.087 | 0.161 |
|  | Volume in the first 2 hrs | 0.002 | -0.147–0.15 | 0.982 | 0.029 | -0.082–0.14 | 0.605 | 0.007 | -0.035–0.05 | 0.738 |
| Septic shock | Abx within 1 hr | 0.314 | 0.02–0.603 | 0.037 | 0.159 | -0.099–0.417 | 0.223 | 0.026 | -0.105–0.158 | 0.685 |
|  | Age (months) | 0.000 | -0.003–0.002 | 0.638 | 0.000 | -0.002–0.002 | 0.894 | 0.002 | 0.001–0.003 | 0.001 |
|  | PIM 3 | 0.308 | -0.19–0.806 | 0.221 | 0.177 | -0.261–0.616 | 0.423 | 0.038 | -0.2–0.276 | 0.747 |
|  | Comorbidities | 0.007 | -0.287–0.301 | 0.963 | -0.066 | -0.326–0.194 | 0.612 | 0.063 | -0.075–0.202 | 0.356 |
|  | Volume in the first 2 hrs | -0.129 | -0.381–0.124 | 0.312 | -0.116 | -0.339–0.106 | 0.300 | -0.022 | -0.137–0.093 | 0.701 |
| Severe Sepsis | Abx within 1 hr | -0.328 | -0.607– -0.049 | 0.022 | -0.074 | -0.257–0.108 | 0.422 | 0.032 | -0.016–0.080 | 0.190 |
|  | Age (months) | 0 | -0.002–0.002 | 0.844 | -0.001 | -0.002–0.000 | 0.162 | 0.000072 | 0.000–0.000 | 0.695 |
|  | PIM 3 | 0.228 | -0.173–0.628 | 0.262 | 0.319 | 0.039–0.598 | 0.026 | -0.061 | -0.133–0.011 | 0.095 |
|  | Comorbidities | -0.122 | -0.354–0.11 | 0.299 | -0.168 | -0.323–-0.012 | 0.035 | 0.006 | -0.033–0.045 | 0.763 |
|  | Volume in the first 2 hrs | 0.093 | -0.092–0.278 | 0.323 | 0.098 | -0.031–0.227 | 0.134 | 0.007 | -0.026–0.041 | 0.675 |

VFD: ventilation-free days, LOS: length of stay, PICU: pediatric intensive care unit, CI: confidence interval, PIM 3: Pediatric Index of Mortality-3
